# Validation of the Adolescent Pediatric Pain Tool for the Multidimensional Measurement of Pain in Children and Adolescents Diagnosed with Osteogenesis Imperfecta

**DOI:** 10.1080/24740527.2019.1626705

**Published:** 2019-07-18

**Authors:** Madalina Boitor, Céline Gélinas, Frank Rauch, Eufemia Jacob, Sylvie LeMay, Jaimie Isabel Carrier, Claudette Bilodeau, Argerie Tsimicalis

**Affiliations:** a Ingram School of Nursing, McGill University, Montreal, Quebec, Canada; b Centre for Nursing Research and Lady Davis Institute, CIUSSS Centre-Ouest-Ile-de-Montréal, Jewish General Hospital, Montréal, Québec, Canada; c Department of Pediatrics, McGill University, Montreal, Quebec, Canada; d Clinical Research, Shriners Hospitals for Children–Canada, Montreal, Quebec, Canada; e UCLA School of Nursing, University of California, Los Angeles, California, USA; f Faculty of Nursing, University of Montreal, Montreal, Quebec, Canada; g CHU Ste-Justine Research Centre, Centre hospitalier universitaire (CHU) Sainte-Justine, Montreal, Quebec, Canada

**Keywords:** Psychometrics, pain assessment, pediatric pain, orthopedic

## Abstract

**Background**: The Adolescent Pediatric Pain Tool (APPT) is a self-reported, multidimensional assessment of pain location, intensity, and quality in children and adolescents. Yet, it has not been validated for use in children and adolescents with osteogenesis imperfecta (OI).

**Aims**: This study aimed to validate and evaluate the feasibility of the APPT for pain assessment in children and adolescents with OI.

**Methods**: A prospective observational study was conducted at a university-affiliated pediatric hospital in Canada. Thirty-three children and adolescents with OI participated by completing the APPT pre-bisphosphonate intravenous infusion and 1 week post-bisphosphonate intravenous infusion. Main outcomes were internal consistency, convergent and discriminative validity, and feasibility.

**Results**: The Kuder-Richardson test of internal consistency was 0.863, 0.661, and 0.729 for the Sensory, Affective, and Evaluative subscales, respectively. For the entire pain quality scale, the Cronbach’s alpha was 0.835. Regarding convergent validity, a moderate correlation was observed between the ratings on the pain intensity scale and the Faces Pain Scale–Revised (Spearman’s rho = 0.711). Patients for whom pain was a problem reported higher pain intensity (Mann Whitney U = 41.50, P = 0.032) and more pain quality descriptors (Mann Whitney U = 45.50, P = 0.020) and painful body areas (Mann-Whitney U = 25.50, P = 0.001) than those for whom it was not (Mann-Whitney U, P < 0.05). In terms of feasibility, completing the tool may require a considerable time commitment and assistance from a clinician or parent, especially if the patient is experiencing pain and provides detailed pain location and quality information by completing the APPT.

**Conclusions**: This study suggests that the APPT is valid for the multidimensional assessment of pain in children and adolescents with OI, but feasibility needs to be enhanced.

## RÉSUMÉ

**Contexte**: L’outil d’évaluation de la douleur des adolescents en pédiatrie (APPT) est une évaluation multidimensionnelle auto-déclarée de l’emplacement, de l’intensité et de la qualité de la douleur chez les enfants et les adolescents. Toutefois, l’utilisation de cet outil auprès des enfants et des adolescents souffrant d’ostéogénèse imparfaite n’a pas encore été validée.

**But**: Cette étude avait pour but de valider et d’évaluer la faisabilité de l’APPT pour l’évaluation de la douleur dans cette population.

**Méthodes**: Une étude observationnelle prospective a été menée dans un centre hospitalier universitaire à vocation pédiatrique au Canada. Trente-trois enfants et adolescents atteints d’ostéogénèse imparfaite ont participé à cette étude en répondant à l’APPT avant l’infusion intraveineuse de bisphosphonate et une semaine après celle-ci. Les principaux résultats étaient la cohérence interne, la validation convergente et discriminante, et la faisabilité.

**Résultats**: Le résultat du test de Kuder-Richardson pour mesurer la cohérence interne était de 0,863 pour le sensoriel, 0,661 pour l’affectif et 0,729 pour les sous-échelles d’évaluation. Pour l’échelle de qualité de la douleur au complet, l’indice alpha de Cronbach était de 0,835. En ce qui concerne la validation convergente, une corrélation modérée a été observée entre les scores obtenus à l’échelle d’intensité de la douleur et le FPS-R (Rho de Spearman= 0,711). Les patients pour lesquels la douleur était problématique ont fait état d’une intensité de la douleur plus élevée, ont eu recours à un plus grand nombre de descripteurs de la qualité de la douleur et ont identifié davantage de parties du corps douloureuses que ceux pour lesquels la douleur n’était pas problématique (Mann-Whitney U, *p* < 0,05). En ce qui concerne la faisabilité, le temps requis pour répondre au questionnaire peut être considérable et l’aide d’un clinicien ou d’un parent peut être nécessaire, particulièrement si le patient ressent de la douleur et donne de l’information détaillée sur la localisation et la qualité de la douleur en répondant à l’APPT.

**Conclusions**: Selon cette étude, l’APPT est valide pour l’évaluation multidimensionnelle de la douleur chez les enfants et les adolescens souffrant d’oastéogénèse imparfaite, mais sa faisabilité doit être améliorée.

## Introduction

Osteogenesis imperfecta (OI) is a rare inherited connective tissue disorder characterized mainly by fractures, bone deformity, and short stature, in addition to the possible involvement of other organ systems.^1^ Though there is no cure for OI, clinical management involves rehabilitation, occupational and physical therapy, orthopedic surgery, and pharmacological therapy.^2,3^ Bisphosphonate therapy is the most widely used pharmacological treatment shown to increase the areal bone mineral density.^4–6^ However, long-bone fractures are still frequent.^3^ Acute pain is mainly fracture related and reaches moderate to severe intensity in most children with OI, whereas nonfracture pain is less intense and experienced 50% of time.^7^ Regardless of intensity, pain is present, complex in quality, and located in several sites in children and adolescents with OI.^8^

Recommended by PedIMMPACT,^9^ the Adolescent Pediatric Pain Tool (APPT) is a self-report, multidimensional measure of pain for use in children (8–12 years) and adolescents 13 years and older.^10^ Several studies have established the validity and reliability of the APPT for use with children and adolescents who (1) are healthy^11,12^; (2) have an acute (e.g., postsurgery)^11–15^ condition; or (3) have a chronic conditions (e.g., cancer, sickle cell disease).^16–19^ However, the validity and reliability of the APPT for use with children and adolescents with OI has yet to be established. Indeed, reliability and validity are not fixed properties of a measurement tool but are targeted to its use with a specific patient population and within a specified context. Internal consistency is a measure of reliability that verifies whether all domains of a scale measure the same construct. Validity refers to the conclusions that can be drawn from the interpretation of scores of a scale and serves to ensure that the tool is measuring what is actually intended.^20^

This study aimed to validate the use of the APPT for the assessment of pain in children and adolescents with OI by examining internal consistency and convergent and discriminative validity and describe its feasibility.

## Materials and Methods

### Design, Setting, and Sample

A prospective observational design was used. This study was conducted at Shriners Hospitals for Children–Canada, Montreal. Patients were eligible if they were between the ages of 8 and 21 years, diagnosed with OI, receiving intravenous bisphosphonates at the study site, able to speak or read French/English, and not pregnant. The sample size was calculated for use in another study using the same participants to provide preliminary pain and quality of life data^21^ and was sufficient to reach strong correlations and significant Mann-Whitney test results. The proposed sample size was considered appropriate given the rare occurrence of OI and feasibility constraints.

### Procedures

Recruitment took place the day children and adolescents were attending their hospital visit as in- or outpatient. The clinical team providing care to patients shared the contact information of interested eligible patients with the research nurse. A research nurse approached parents for participation, provided information regarding the study, and obtained written informed parental consent and child assent. Patients aged 18 and over provided written consent themselves.

Demographic (i.e., age, gender, ethnicity) and clinical data (i.e., OI type) were collected by a research nurse. Those willing to participate completed the APPT,^10^ the Faces Pain Scale–Revised (FPS-R),^22^ and the Pediatric Quality of Life 4.0 Generic Core Scales (PedsQL4)^23^ in their hospital room before the start of the bisphosphonate intravenous infusion. A research nurse was present in the room in the event that the participant needed help to complete the instruments. These data were used to test the reliability and validity of the APPT with children and adolescents with OI.

With telephone support, provided by the research nurse, the APPT was completed again 1 week post-bisphosphonate intravenous infusion while participants were at home. The research nurse provided participants with a maximum of three telephone reminders and a reminder letter by mail to favor timely data collection. These data were used to describe the feasibility of completing the APPT in the home.

### Instruments

#### The Adolescent Pediatric Pain Tool

The APPT is a one-page, double-sided, self-report measure of pain for use in children and adolescents.^10^ It has three subscale scores measuring the different dimensions of pain. First, pain location is indicated on a segmented body outline diagram (BOD) so that the number of pain sites may be counted.^14^ Second, pain intensity is indicated on a 10-cm line, called a word graphic rating scale (WGRS), on a scale including *no pain, little, medium, large*, and *worst possible pain*.^12^ Third, *pain quality is* quantified on the pain rating index scores for the sensory, affective, evaluative, and temporal aspects of pain.^11,24–26^ The Sensory subscale has 37 items, the Affective subscale has 11, and the Evaluative subscale has eight. The temporal aspects were not available in French and were not measured in our population.

The reliability and validity of the APPT have been tested with various populations. The interrater reliability of the body outline diagram was tested with the medical/surgical pediatric population and nurses, with Cohen’s kappa coefficients ranging from 0.55 to 0.71 for the site number agreement, 0.30 to 0.34 for the location of markers, and 0.36 to 0.47 for the surface area covered.^14^ Test–retest reliability was examined between pain intensity ratings on the WGRS in the morning and those in the afternoon in hospitalized children, with a strong correlation (*r* = 0.91).^12^ Content validity was ensured by testing the body outline diagram in 175 hospitalized children and adolescents between the ages of 8 to 17 years,^14^ and the pain quality descriptors with were tested with 1,223 ethnically diverse community-based or hospitalized children and adolescents.^11^

Discriminative validity has been supported because the Pain Intensity and Pain Quality subscales of the APPT had lower scores over 5 days postoperatively (*n* = 65),^13^ and significantly lower pain intensity was observed after pain treatment compared to prior in children with leukemia (*n* = 95)^17^ and sickle cell disease (*n* = 37).^18^ Convergent validity was supported with moderate to high Pearson’s correlations between the WGRS and a visual analog scale (*r* = 0.82), graded graphic scale (*r* = 0.90), numeric scale (*r* = 0.78), and color scale (*r* = 0.68) in healthy and medical/surgical child patients (*n* = 1223).^12^ Confirmatory factor analysis indicated factor loadings of 0.71, 0.49–0.59, and 0.59–0.85 for the Evaluative, Sensory, and Affective word list subscales, respectively.^11^

#### Self-Report of Pain Intensity

The FPS-R^22^ was used to assess the intensity of pain. This tool, developed specifically for children aged 4–18 years, is used as a standard of clinical care at the study site. The FPS-R consists of six faces depicting expressions ranging from *no pain* to *most pain possible*, placed at equal intervals horizontally on a 0–10 numeric pain intensity scale. Test–retest reliability and content and construct validity have been well established.^27^

#### Self-Report of the Extent to which Pain was a Problem

The pain item of the PedsQL4 was used to test discriminative validity. The PedsQL4 includes 23 items and was validated for measuring health-related quality of life in healthy and acutely and chronically ill children and adolescents aged 2 to 18 years.^23,28,29^ A 5-point response scale is utilized across each item (0 = *never*, 1 = *almost never*, 2 = *sometimes*, 3 = *often*, and 4 = *almost always a problem*). The pain item measures the extent to which pain is a problem for children and adolescents with OI. For this study, the pain item was dichotomized to indicate whether pain was not a problem (i.e., 0 or 1) or was a problem (i.e., 2, 3, or 4).

### Data Analysis

Descriptive statistics were calculated using SPSS software (version 22). The Kuder-Richardson test of internal consistency was performed using the individual binary items (i.e., present/absent) of the Sensory, Affective, and Evaluative Pain Quality subscales and Cronbach’s alpha for the entire Pain Quality Scale. The Kuder-Richardson test is the appropriate statistical test for binary variables, and Cronbach’s alpha is the test of choice for items with multiple options such as the Sensory, Affective, and Evaluative subscales. Convergent validity was estimated using Spearman’s rho correlation coefficient, and discriminative validity was estimated using the Mann-Whitney test. Feasibility was assessed by referring to field notes, where the duration of assessment, observations, need for assistance, and any other comments expressed by participants were noted.

## Results

### Sample Characteristics

A total of 36 children were screened and approached for participation in the study. Of these, 34 agreed to participate but only 33 completed the data collection forms before the bisphosphonate treatment. Participants had a median age of 12 (range 8–19), half of them were male (51.5%), and the majority were of Caucasian (84.8%) ethno-cultural backgrounds. Most children and adolescents were diagnosed with type IV OI (*n* = 16), followed by type I (*n* = 8), type III (*n* = 8), and type VII (*n* = 1).

### Report of Pain Prior to Bisphosphonate Intravenous Infusion

**Figure 1.**
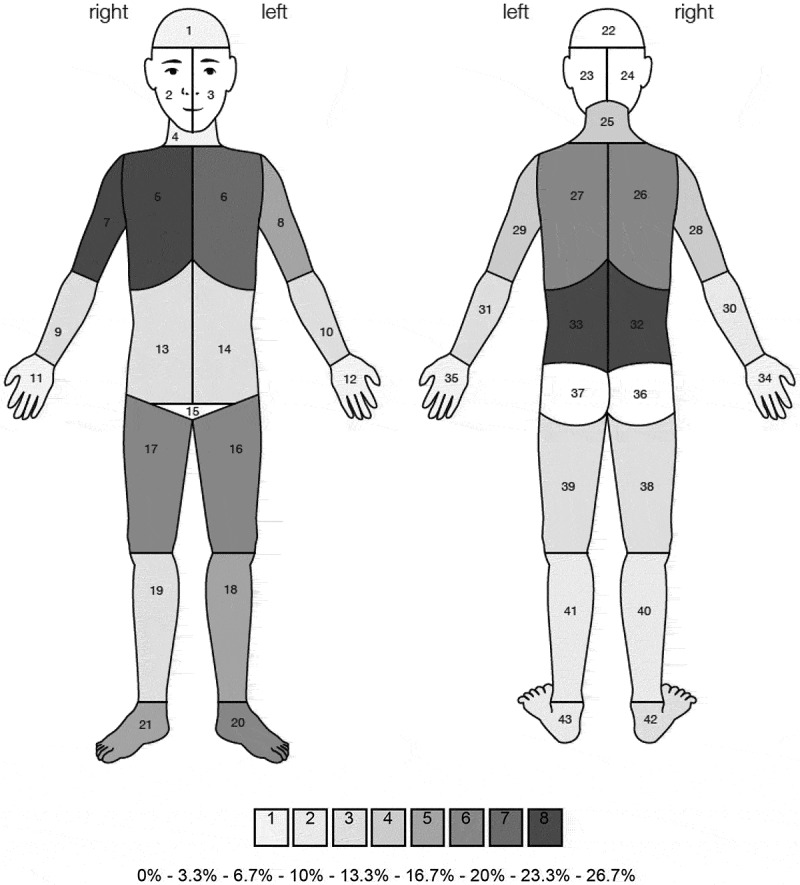
Frequency of pain reports by children and adolescents with osteogenesis imperfecta measured using the body outline diagram (*n* = 33) prior to receiving bisphosphonate treatment.

Children and adolescents reported mild pain intensity before the bisphosphonate treatment (median 23, range 0–76, interquartile range [IQR] 47) as measured with the WGRS and selected a median of two (range 0–29, IQR 5.5) painful body segments that were generally in the proximity of hip, ankle, and shoulder joints (Figure 1). The most common pain quality descriptors were sensory, followed by evaluative and affective ones (Table 1). Pain intensity measured using the FPS-R had a median of 2 (range 0–10, IQR 2). As per the pain item of the PedsQL4, pain did not represent a problem for most children and adolescents with OI (*n* = 25, 76%).

**Table 1.** Frequency of pain quality descriptors of the adolescent pediatric pain tool selected by children and adolescents with osteogenesis imperfecta.

Pain quality descriptor	*n* = 33
Sensory	
Aching	8
Hurting	6
Like an ache	6
Like a hurt	2
Sore	11
Beating	1
Hitting	4
Pounding	2
Punching	0
Throbbing	5
Biting	4
Cutting	1
Like a pin	2
Like a sharp knife	2
Pin like	1
Sharp	3
Stabbing	3
Blistering	1
Burning	1
Hot	2
Cramping	6
Crushing	0
Like a pinch	3
Pinching	2
Pressure	7
Itching	2
Like a scratch	0
Like a sting	0
Scratching	1
Stinging	2
Shocking	2
Shooting	5
Splitting	1
Numb	5
Stiff	7
Swollen	3
Tight	7
Affective	
Awful	4
Deadly	0
Dying	0
Killing	0
Crying	4
Frightening	0
Screaming	2
Terrifying	0
Dizzy	3
Sickening	2
Suffocating	1
Evaluative	
Annoying	13
Bad	3
Horrible	1
Miserable	0
Terrible	1
Uncomfortable	16
Never goes away	3
Uncontrollable	4

### Reliability

#### Internal consistency

The internal consistency of the Sensory, Affective, and Evaluative Pain Quality Descriptor subscales was examined. Although the Sensory subscale includes 37 pain quality descriptors, several were not used by children with OI to describe their pain, such as “punching,” “crushing,” “like a scratch,” and “like a sting.” Similarly, the Affective subscale has 11 descriptors, yet five of them were not selected by children (i.e., “deadly,” “dying,” “killing,” “frightening,” and “terrifying”). Only one (i.e., miserable) of the eight evaluative pain quality descriptors was not used.

The Kuder-Richardson test of internal consistency was 0.863 for the Sensory subscale, 0.661 for the Affective, and 0.729 for the Evaluative subscale. A higher internal consistency for the Affective subscale was obtained if the item “screaming” was removed (Kuder-Richardson–20 = 0.726). This item was reported by only two participants. The internal consistency of the Pain Quality Scale was calculated using the percentage scores for each of these three subscales, yielding a Cronbach’s alpha of 0.835.

The Spearman’s rho interdimension correlation coefficients were moderate to high, ranging from 0.632 to 0.807.^30^ The highest interdimension correlation was between pain intensity ratings and total number of pain quality descriptors, whereas the lowest was between pain intensity ratings and total number of body areas.

### Validity

#### Convergent Validity

Convergent validity was used to assess the validity of the use of the APPT with other related measure of pain. A moderate correlation was observed between the pain intensity ratings on the WGRS and FPS-R (Spearman’s rho = 0.711).

#### Discriminative Validity

Discriminative validity was used to examine the ability of the APPT to discriminate between two groups of children and adolescents with OI: those for whom pain was never or almost never a problem (i.e., 0 or 1; *n* = 25) and those for whom pain was sometimes to almost always a problem (i.e., 2, 3, or 4; *n* = 8). The WGRS could discriminate these two groups with higher pain intensities reported by those for whom pain was a problem compared to those for whom it was not (Mann Whitney *U* = 41.50, *P* = 0.032). Similarly, children and adolescents with OI reported significantly more pain quality descriptors (Mann Whitney *U* = 45.50, *P* = 0.020) and painful body areas (Mann-Whitney *U* = 25.50, *P* = 0.001) if pain was a problem compared to pain not being a problem.

### Feasibility

The APPT was completed by 33 children and adolescents in the hospital before receiving their bisphosphonate treatment. Adolescents completed the APPT by themselves, and children under 13 years required assistance from a research nurse to complete the tool. The research nurses assisted them by reading the questions and presenting the possible answers. Completing the entire APPT took 10 min if participants reported no pain and up to 30 min if participants experienced pain. Children were easily distracted while responding to questions and occasionally required the research nurse to redirect their attention to the content of the APPT. While at home, 28 (85%) children and adolescents completed the APPT with support over the telephone as needed, which took more time than providing in-person support. Younger children were more likely to report that completing the APPT was time-consuming.

## Discussion

The use of multidimensional scales such as the APPT has been recommended in clinical practice to capture the diverse dimensions of pain.^9^ To date, few studies have assessed pain using a multidimensional measure as a primary or secondary outcome in OI research.^7,8^ Indeed, accurate assessment of pain is contingent on using measures that have been validated with the target population and in the specific context in which it will be implemented. The present study offers preliminary data to support the reliability (i.e., internal consistency) and validity (i.e., convergent and discriminative) of the APPT for pain assessment in children and adolescents with OI whose pain is present, problematic, and underresearched.^8^ Clinicians may use the APPT to guide a multidimensional pain assessment, and researchers may use it as a primary or secondary outcome in research.

Recognizing that this study included a small sample of children, the study findings do support the internal consistency and convergent and discriminative validity of the use of the APPT for the assessment of pain in children and adolescents with OI, but additional research is needed. Reliability analyses indicated acceptable internal consistency^20^ for the overall Pain Quality Scale as well as the Sensory and Evaluative subscales. The Affective subscale reached acceptable internal consistency level if the item “screaming,” which was only endorsed by two participants, was removed. Of note, not all descriptors were used by participants to indicate their quality of pain; warranting further inquiry. This could be partly due to timing, context, or small sample size but may also suggest that children and adolescents with OI do not perceive their pain to be severe enough to be called killing, terrifying, or miserable, especially because they self-reported mild pain intensity on the WGRS. Instead, the quality of pain in the context of OI seems to coincide with descriptors such as “uncomfortable,” “annoying,” and “sore,” as documented in previous studies.^7,8^ The timing of assessment may have also influenced the choice of descriptors because fracture pain was not captured in the present study. Zack et al.^7^ reported that children and adolescents most often used the words *uncomfortable* or *throbbing* to describe their fracture pain, which is accompanied by a higher pain intensity. The children and adolescents also reported fracture pain ranging from 2 to 100 mm on a 100-mm scale with mean of 66.0 mm (SD 28.5) and nonfracture pain ranged from 12 to 99 mm with a mean of 47.0 mm (SD 27.5).

Consistent with previous validation studies conducted with cancer^31^ and orthopedic pediatric populations,^15^ convergent validity was supported because pain intensity scores on the WGRS correlated moderately with scores obtained on the 0–10 FPS-R. Opportunity for ongoing validation of pain intensity may be possible with the use of “Portrait-Bobo,” a new pain tool developed and piloted by Zabalia and Mancel to score pain intensity and emotions associated with OI.^32^

All three dimensions of the APPT (i.e., intensity, quality, and location) could discriminate children and adolescents with OI for whom pain was a problem versus those for whom it was not, thereby providing support for the construct validity for the use of the APPT with this population. Regarding construct validity in the surgical context, pain recorded on the Pain Intensity Scale of the APPT was shown to decrease gradually postoperatively and demonstrated sensitivity to changes in pain levels over time.^12^ Opportunity for ongoing validation is warranted and may include discriminating between individual pain experiences (e.g., fracture and nonfracture pain, during restrictive and nonrestrictive mobility),^8^ responsiveness to treatments, and the inclusion of the French-speaking among other non-English- or Spanish-speaking childhood OI populations.

The use of the APPT allows for a comprehensive assessment of the pain experience of children and adolescents with OI by eliciting their evaluation of pain intensity, quality, and location; however, completing the tool may require a considerable time commitment and assistance from a clinician or parent, especially if the child is experiencing pain. Making the APPT more attractive and visually appealing through its use in an electronic format could enhance its feasibility in both the hospital and home. The use of pain assessment tools in an electronic format has been shown to be preferred to the traditional paper format by children as young as 4 years old^33^ and to be feasible in a pediatric population with increased compliance, including absence of errors and omissions due to electronic programming of the e-tool.^34^ The design of multidimensional pain assessment tools in a game-based smartphone application with a virtual reward system has been shown to be appealing, easy to use, and not bothersome to complete for children and adolescents with cancer.^35^

Usability and feasibility testing of the web-based electronic version of the APPT, accessed by using a smartphone, tablet, or other handheld electronic device, was previously conducted for use in children and adolescents with sickle cell disease.^36,37^ These studies support the use of these technologies to improve the self-reporting of symptoms and communication between patients with sickle cell disease and their health care providers, given that they were easy to use and efficient to complete. Future studies are needed to continue validating the use of the APPT in research and practice, evaluate the feasibility of an electronic version of the APPT with children and adolescents with OI, and evaluate their satisfaction with its use for monitoring pain.

### Limitations

This study presented results regarding three subscales of the APPT, including its impact on the feasibility of completing this tool in clinical practice. At the time of the study, the temporal dimension of the APPT was not available in French, and the linguistic validation of a scale warrants a different methodology, which fell outside the scope of this ethically approved study. Now, with multidimensional pain data collected as a primary outcome with the OI population, along with data suggesting that pain is present, future research needs to include the validation of the APPT for the French-speaking population along with the inclusion of the Temporal subscale for validation with the pediatric OI population. A sample size of 33 for validation of the APPT is small, which limits the findings. In the 23 APPT studies reviewed,^38^ sample sizes ranged from one to 351 with an average of 74 participants. In the present study, only eight of the 33 children (24%) reported bothersome pain levels (sometimes, often, almost always a problem), which is an extremely small number for validating a pain scale. However, this number also suggests that 24% of children with OI are reporting pain, which may be clinically significant and warrants further intervention. A larger sample size is needed to further test these estimates and contribute to the ongoing reliability and validity of the APPT. With a larger sample size, a factor analysis will be permissible along with the identification of the number of dimensions comprising the APPT. The feasibility of scoring with the APPT was only assessed via observation and documentation of field notes. Future studies could explore children and adolescents’ perceptions of completing this tool to track their pain and their suggestions for improving its ease of use by conducting brief individual semistructured interviews.

### Conclusion

This study offers preliminary data to support that the APPT is valid for the multidimensional assessment of pain in children and adolescents with OI. Though the APPT allowed the pediatric OI population to elaborate on their pain experiences, completing the tool could be time-consuming, especially for children experiencing pain. To enhance the feasibility of completing the APPT, more work is needed to increase its attractiveness and ease of scoring, possibly by using an interactive electronic format.
